# Reproducibility of radiomics for deciphering tumor phenotype with imaging

**DOI:** 10.1038/srep23428

**Published:** 2016-03-24

**Authors:** Binsheng Zhao, Yongqiang Tan, Wei-Yann Tsai, Jing Qi, Chuanmiao Xie, Lin Lu, Lawrence H. Schwartz

**Affiliations:** 1 Department of Radiology, Columbia University Medical Center, 710 West 168^th^ Street, New York NY 10032, USA; 2 Department of Biostatistics, Columbia University Medical Center, 722 West 168^th^ Street, New York NY 10032, USA.

## Abstract

Radiomics (radiogenomics) characterizes tumor phenotypes based on quantitative image features derived from routine radiologic imaging to improve cancer diagnosis, prognosis, prediction and response to therapy. Although radiomic features must be reproducible to qualify as biomarkers for clinical care, little is known about how routine imaging acquisition techniques/parameters affect reproducibility. To begin to fill this knowledge gap, we assessed the reproducibility of a comprehensive, commonly-used set of radiomic features using a unique, same-day repeat computed tomography data set from lung cancer patients. Each scan was reconstructed at 6 imaging settings, varying slice thicknesses (1.25 mm, 2.5 mm and 5 mm) and reconstruction algorithms (sharp, smooth). Reproducibility was assessed using the repeat scans reconstructed at identical imaging setting (6 settings in total). In separate analyses, we explored differences in radiomic features due to different imaging parameters by assessing the agreement of these radiomic features extracted from the repeat scans reconstructed at the same slice thickness but different algorithms (3 settings in total). Our data suggest that radiomic features are reproducible over a wide range of imaging settings. However, smooth and sharp reconstruction algorithms should not be used interchangeably. These findings will raise awareness of the importance of properly setting imaging acquisition parameters in radiomics/radiogenomics research.

Radiogenomics (radiomics) is a rapidly emerging field, the goal of which is to determine the association between a cancer’s genotype and imaging phenotype^1^,^2^,^3^,^4^. Unlike other molecular characterizations that are based on genomic technologies, the radiomic approach strives to develop and use quantitative methods (quantitative image features) to characterize phenotypic differences of cancers and/or cancer subtypes, and to do so based on noninvasive radiologic images that can be acquired during routine clinical practice. Recent studies on qualitative as well as quantitative assessments of tumor characteristics suggest that cancer imaging phenotypes captured by computed tomography (CT), positron emission tomography (PET) and magnetic resonance imaging (MRI) can reveal the underlying gene expression profiles in many cancer types such as hepatocellular carcinoma (HCC), glioblastomas (GBM), lung cancer, esophageal cancer and renal cancer^5^,^6^,^7^,^8^,^9^,^10^,^11^,^12^. With the increasing use, in cancer, of precision medicine–an emerging approach to disease treatment and prevention that takes into account individual variability in genes, environment, and lifestyle for each person–and with the recognition of tumor genotype heterogeneity, a non-invasive imaging tool to help characterize tumors would be of tremendous value.

For a quantitative image feature to serve as a biomarker for tumor phenotype and thus to aid in cancer diagnosis, prognosis, response prediction and assessment of therapy, it must be reproducible, i.e., its value should stay unchanged or minimally changed when the feature is computed from a repeat scan acquired after a short time interval^13^,^14^. Image features with higher reproducibility will have the potential to distinguish smaller differences between different tumor phenotypes. In current clinical practice, a wide variety of scanning techniques and parameters are used. Despite the fact that quantitative radiomic features are computed from a set of image elements (an image element is a physical point in a raster image) and thus can be sensitive to, for example, image spatial resolution (image element size) and noise (random variation of image element value) that are governed by the scanning techniques and parameters, little attention has been given to date to the acquisition of scans such as CT scans, for the purpose of determining how scan acquisition techniques and parameters affect the reproducibility of quantitative radiomic features^15^,^16^.

Figure 1 shows an example of a lung cancer tumor captured on a CT scan that was reconstructed using different imaging parameters of three slice thicknesses (1.25 mm, 2.5 mm and 5 mm) and two reconstruction algorithms (one sharp and one smooth algorithm). We can see that tumor heterogeneity (brightness details or textures) are better depicted on the sharper images than on the smoother ones given the same slice thickness (e.g., Fig. 1(a,b)). If the effect of such imaging acquisition variables on radiomic features is unknown, then the opportunity to identify reliable and meaningful radiomic features that can accurately characterize tumor phenotypes may be missed and study findings may not be reproducible.

Using a uniquely acquired same-day repeat CT lung cancer dataset, with each scan reconstructed at one of the six different imaging settings as discussed in Fig. 1, we explored the reproducibility of radiomic features over a wide range of CT imaging settings used in clinical practice and clinical trials. In separate analyses, we studied the agreement of radiomic features when computed from repeat CT scans reconstructed using different imaging settings.

## Methods

### Lung Cancer Image Data

In this study we used an already-acquired de-identified image data set from 32 lung cancer patients. The data had been collected during a previous IRB-approved study in which patient informed consent was waived. It was a HIPAA-compliant, same-day repeat CT study (ClinicalTrials.gov identifier NCT00579852)^17^. In that study, each patient underwent two unenhanced CT scans within 15 minutes. During data collection, each of a patient’s two repeat scans was reconstructed into 6 image series that were combinations of three slice thicknesses (1.25 mm, 2.5 mm and 5 mm) and two reconstruction algorithms (lung (L) and standard (S)). The 6 corresponding imaging settings are referred to as: 1.25L (i.e., 1.25 mm slice thickness and lung reconstruction algorithm), 1.25 S, 2.5 L, 2.5 S, 5 L and 5 S. The imaging protocol is provided in the Supplementary Tables section (Table S-1).

One of the 6 combinations of image series and imaging settings, the 1.25 mm slice thickness and lung reconstruction series (1.25 L), later became publicly accessible through The Reference Image Database to Evaluate Therapy Response (RIDER) project sponsored by the National Cancer Institute and has been used worldwide to study the reproducibility of tumor size measurements and, more recently, to study radiomic features^14^,^17^,^18^,^19^,^20^,^21^.

### Lung Tumor Segmentation

Tumor segmentation was performed using a home-grown, semi-automated algorithm developed for lung tumors on CT images^22^. Three experienced radiologists independently segmented the 32 tumors (one tumor per patient) on all 12 image series (2 repeat scans × 6 imaging settings/scan) in 12 reading sessions. The time interval between any two sessions was two to three weeks to reduce the effects of memory on the radiologists’ readings. If any computer-generated segmentation result was suboptimal, a radiologist was allowed to modify the result (i.e., contour) using the editing tool, also developed in-house. The final segmentation result was based on the common tumor volumes agreed to by 2 of the 3 radiologists.

### Quantitative Image Features

From each segmented tumor, we extracted a set of well-defined quantitative image features that describe tumor size, shape, margin spiculation and sharpness, and density distributions without spatial information (histogram-derived, first-order density statistics) and with spatial information (texture patterns). Tumor size was measured using (i) the maximal diameter (Uni), (ii) the product of the maximal diameter and its maximal perpendicular diameter (Bi), and (iii) volume. Shape features included Compactness Factor, Roundness Factor, Eccentricity, and Solidity. Surface shape was described by the Shape Index and density change across the tumor boundary was quantified by Sigmoid Functions. First-order statistical features included Mean, Standard Deviation, Skewness and Kurtosis of tumor densities. Texture features included the Gray-Level Co-occurrence Matrix (GLCM), Gray Tone Difference Matrix (GTDM), Spatial Correlation, Rung Length, Laws Energy and Edge Frequency. There were also multi-scale texture features such as Gabor Energy, Wavelets and the Laplacian of the Gaussian (LoG), and a model-based feature of Fractal Dimension.

In total, we extracted 89 radiomic features that were grouped into 15 feature classes. The definitions and formulas of these features and relevant references are provided in the Supplementary Methods section.

### Statistical Analysis

To study the reproducibility of radiomic features under a wide range of imaging parameter settings, the following 6 combinations of repeat scans (the first scan versus the second scan) were included: 1.25L vs 1.25L, 1.25S vs 1.25S, 2.5L vs 2.5L, 2.5S vs 2.5S, 5L vs 5L, and 5S vs 5S. In separate analyses, we explored measurement agreement of radiomic features when calculated for the repeat scans but reconstructed using different imaging parameters. Because of the large number of possible combinations, the different imaging settings between repeat scans were limited to the following three: 1.25L vs 1.25S, 2.5L vs 2.5S, and 5L vs 5S, i.e., same slice thickness but different reconstruction algorithms

In this work, concordance correlation coefficients (CCCs) as defined by Lin were employed to study the reproducibility of radiomic features^23^. The analysis was done with R version 2.15.2^24^. A CCC value of 0.85 was used as the threshold to assess reproducibility, i.e., a radiomic feature with CCC ≥ 0.85 was considered a reproducible feature.

## Results

### Tumor Segmentation

**Figure 2:**
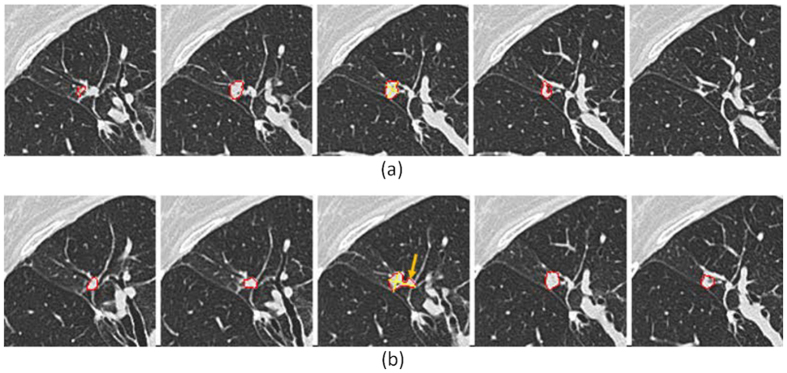
Inconsistency in tumor segmentation on repeat CT scans. This example shows a small tumor (tumor #17; 10.7 mm in diameter) in the vicinity of blood vessels. Segmentation results (tumor contours in red) are superimposed on the original images. Only the tumor was delineated on the first scan (**a**), but a part of the surrounding vessels was included along with the tumor on the repeat scan images (arrow) (**b**). The imaging setting was 1.25mm slice thickness and lung reconstruction (1.25L)

Prior to the extraction of quantitative radiomic features, the tumor needs to be segmented. One issue we want to address before presenting our reproducibility results is that of variability in tumor segmentation. To reduce segmentation-related variability, in this work, each tumor on the two repeat scans was delineated independently by three radiologists. However, we still found considerable segmentation-based inconsistency in a small tumor that was in the vicinity of blood vessels (Fig. 2). Two radiologists consistently either included or excluded the part of the surrounding vessels as the tumor when blindly segmenting it on the two repeat scans. The third radiologist, however, made an inconsistent decision. This radiologist excluded the vessel part from the tumor on the first scan but considered it as part of the tumor on the repeat scan two weeks later. As the result, only the tumor was delineated on the first scan (Fig. 2a), but a part of the surrounding vessels was included along with the tumor on the repeat scan (arrow in Fig. 2b).

**Figure 3:**
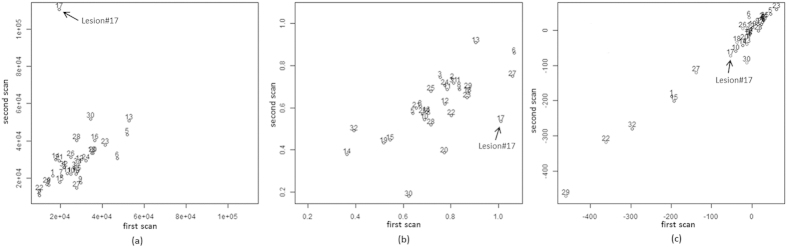
CCC plots of three example image features before removing the outlier tumor. After removing tumor #17, the CCC values changed from (**a**) 0.28 to 0.76 for Wavelet_DWT_LD at 5S, and (**b**) 0.44 to 0.53 for Roundness-Factor_2D at 1.25L. The CCC value remained unchanged (0.98) for Density_Mean at 1.25L (**c**).

Figure 3 shows the CCC plots of three example radiomic features before the removal of the small tumor #17. Different degrees of change in CCC values before and after removing this small tumor from the analysis were observed, implying different effects a segmentation inconsistency/error can have on different quantitative features of a tumor. Due to the small tumor size at the second level of Wavelets decomposition at the imaging setting of 5S, this feature was largely affected by the tumor segmentation inconsistency (tumor #17 was a significant outlier by Dixon’s Q-test result^25^) (Fig. 3a). However, because of the same or similar density values of the tumor and the vessel part considered as the tumor, the segmentation inconsistency had almost no effect on the Mean density (Fig. 3c). The segmentation inconsistency had an effect on the shape feature of Roundness Factor, but no outlier tumor was found in Fig. 3b by Dixon’s Q-test. To further minimize the effect of tumor segmentation, in the following presentation we only report the reproducibility results based on the outlier (tumor #17) removed cohort of 31 tumors.

### Reproducibility of Radiomic Features on Repeat Scans Reconstructed at Identical Imaging Setting

**Figure 4:**
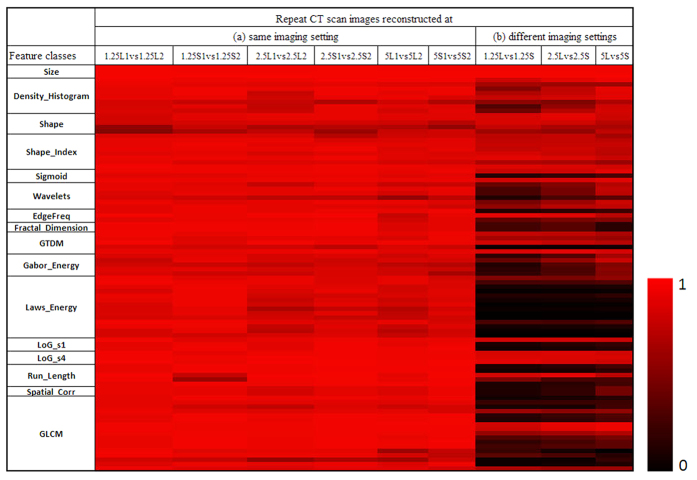
CCC heat map of radiomic features. The CCCs (0 to 1) of the studied radiomic features were computed from repeat CT images reconstructed at (**a**) six identical imaging settings or (**b**) three different imaging settings. There were 89 quantitative features grouped into 15 feature classes. The brighter the red color, the higher the CCC value (i.e., the more reproducibility) of a feature computed for the repeat scans. The label of “1.25L1 vs 1.25L2” means both first and second scans were reconstructed at 1.25mm slice thickness using the lung algorithm. “2.5L vs 2.5S” means both scans were reconstructed at 2.5mm slice thickness but using different algorithms (i.e., lung vs. standard algorithms).

Figure 4 is a heat map with Part (a) showing CCCs for 89 radiomic features computed for repeat CT scans reconstructed at six identical imaging settings. In the heat map, the brighter the red color, the higher the CCC value (i.e., the higher the reproducibility). The corresponding numerical CCC values of these radiomics features are provided in part (a) in Table S-2 in the Supplementary Tables section.

In Part (a) in Table S-2 in Supplementary Tables section, the CCCs of the radiomic features in the feature classes of Size, 3D Density Statistical Features (except Kurtosis at 2.5L, CCC = 0.81), Sigmoid Functions, Edge Frequency (except Coarseness at 5L, CCC = 0.81) and LoG were all greater than 0.85 at all imaging settings, demonstrating the high reproducibility of these features/feature classes over a wide range of imaging reconstruction parameters. We compared a shape feature and the histogram-derived density statistical features computed from 2D and 3D images and found that the 3D features were more reproducible than 2D features across all imaging settings. The 3D shape feature we compared was Compactness Factor, which determined sphericity of a 3D object based on the object’s volume and surface, whereas the complementing 2D shape feature was Roundness Factor, determining the circularity of a 2D object based on its area and perimeter. At 1.25 L, the CCCs of the 3D Compactness Factor and 2D Roundness Factor were 0.86 and 0.53 (p = 0.001). The CCCs of the histogram-derived Density-Mean, Standard Deviation (SD), Skewness and Kurtosis were all higher when calculated in 3D than in 2D, though only the Mean and SD showed statistical significance (p = 0.013, 0.014). For the LoG features, s = 1 indicated no preprocessing and s = 4 indicated that a large Gaussian kernel with σ = 2.5 was applied to preprocess (i.e., to smooth) the images. For all 6 imaging settings, all LoG features (except Uniformity at 1.25 S) were more reproducible when calculated on smoothed images. In the GLCM class, all features had CCC ≥ 0.85 at 1.25L and 13 out of 17 features showed CCC ≥ 0.85 at all 6 imaging settings.

We then considered several alternative CCC thresholds (cut-offs) to define the radiomic features’ reproducibility. Table 1 shows the number and in parentheses the percentage of the radiomic features that had CCC values greater than or equal to a given threshold at each imaging setting. When using a CCC value of 0.85 as the cut-off for reproducibility, the imaging setting of 1.25 L produced the highest number of radiomic features that were considered reproducible. The next best settings were 2.5S and 1.25 S. The imaging setting of 5 L was the least reproducible. When choosing a higher CCC value of 0.90 or 0.95 as the cut-off, the imaging setting of 1.25 S performed the best, followed by 2.5 S and 1.25 L. Again, 5 L performed the worst.

### Measurement Agreement of Radiomic Features on Repeat Scan Images Reconstructed at Different Imaging Settings

Part (b) in the heat map of Fig. 4 shows CCCs for the 89 radiomic features computed on repeat CT scans, like Part (a), but reconstructed at different imaging settings: using a sharp (i.e., lung) or a smooth (i.e., standard) algorithm at three different slice thicknesses. The corresponding CCC values are provided in part (b) in Table S-2 in the Supplementary Tables section. Only morphological features such as Uni, Bi and Volume, some shape-related features (e.g., 3D Compact-Factor, Shape-Index 9), Density Mean, and texture features of the LoG class computed using the large Gaussian Kernel (s = 4) had high CCC values (and bright colors in part (b) in Fig. 4) at all slice thickness levels, indicating their insensitivity to imaging reconstruction algorithms (i.e., image sharpness). In contrast, the majority of texture features, starting with the Wavelets class in part (b) of Table S-2 in the Supplementary Tables section, showed low CCC values (and dark colors in part (b) in Fig. 4) at all slice thicknesses. This indicated that there were big differences in the values of radiomic features when computed on repeat CT scans that were reconstructed using different imaging reconstruction algorithms (here smooth and sharp algorithms).

## Discussion

A recent series of publications has been reporting the value and the potential of quantitative radiographic image features for linking tumor phenotypes to genotypes. Their findings were mostly based on retrospective analysis of imaging data from historical studies and from some clinical trials that were actually not designed for quantitative feature analysis of tumors. Many of the patients studied were on clinical trials where simple, unidimensional tumor measurements were recorded, and, therefore, the precise CT acquisition parameters were not critical. Because these data sets are now being analyzed retrospectively and new data sets are being acquired prospectively, the relative importance of standardization of CT acquisition methods needs to be determined, which was the aim of our study.

It has been well understood and accepted that thinner slice images are more appropriate than thicker ones for measuring tumor volumes and volume changes over time^26^,^27^. However, this may not hold true for other image features, especially texture features. For example, increased noise levels associated with thinner slice images may disturb texture features as many texture features are quite sensitive to fluctuation of image densities. On the other hand, although thicker slices decrease noise levels, they can blur the images (diminish texture details) due to poor spatial resolution along the axial direction and larger partial volume effects. Moreover, at the same slice thickness, a smoother reconstruction algorithm can reduce more noise from images than a sharper one, but the smoother algorithm may hold back useful texture details from images. With the aim of providing some fundamental data that could accelerate radiogenomic research, we attempted, in this work, to gain insight into the proper imaging acquisition settings that would allow us to obtain the most reliable and meaningful radiomic features of lung cancer tumors.

Overall, we found that radiomic features of tumors were reproducible over a wide range of CT imaging parameter settings currently used in clinical care and in clinical trials. Non- texture features and feature classes such as tumor size, histogram-derived density statistics, shape and surface shape were highly reproducible for all imaging settings. Texture features extracted from smoothed images (e.g., LoG with a larger Gaussian kernel) were also very reproducible for all settings. In general, imaging settings of thinner slice thickness (1.25 mm and 2.5 mm) and smoother reconstruction algorithm are more favorable for reproducibly extracting quantitative features. Among the imaging settings, the thick and sharp imaging setting of 5 L fared the worst at the task of reproducibly computing image features.

Most clinical correlation studies use 2D image features. One reason is that they are easy to define and/or compute. However, it is wrong to assume that a tumor can always be characterized by a single image. 2D features are not able to accurately describe the heterogeneity of an entire tumor. Recent studies have begun to report better quantification of tumor heterogeneity and better correlation with clinical outcomes using 3D radiomic features such as Entropy and Uniformity^28^. In our work, we calculated several radiomic features, i.e., size, shape and histogram-related density distributions, in both 2D and 3D, and compared the reproducibility of these features. We found that, in general, radiomic features implemented in 2D were less reproducible than those implemented in 3D. Fewer tumor pixels in a 2D image likely makes the radiomic features more sensitive to different orientations and alignments of images between repeat scans. However, if the image spatial resolution along the z-axis is much lower than that in the x/y axes, the usefulness of the 3D features will drop due to larger partial volume artifacts along the z-direction.

In addition to studying the reproducibility of radiomic features, we explored agreement among the image features using the same repeat scan data but reconstructed at different imaging settings. Due to the already large volume of data in this paper, we only reported the repeat scan images with the same slice thickness but different reconstruction algorithms. Using different reconstruction algorithms, our data show low CCCs for almost all radiomic features at all slice thicknesses. The exceptions were non-texture features (e.g., size, shape and density Mean) as they are less dependent or not dependent on density spatial distribution, and thus are less affected by the reconstruction algorithms, i.e., image sharpness. In contrast, almost all texture features were strongly affected by imaging reconstruction algorithms due to the dependency of texture features on image spatial and density resolutions. It is important to note that texture features are more sensitive to imaging reconstruction parameters than are other features. Therefore, the sharp and smooth reconstruction algorithms should not be used interchangeably in radiogenomics research.

Our study has its limitations. First of all, due to the nature of human studies, we were only able to investigate a single type scanner of one CT vendor. There are many other variables during CT imaging acquisition that were not studied. For example, we did not study the effects of changing other CT instrument settings (e.g., kVp, mAs, pitch), which can also considerably affect image quality, and thus potentially influence the numerical values of radiomic features. Comparison studies on such variables have to be carried out using phantom data. Second, segmentation is a well-known source of variation for tumor volume measurements. The effect on quantitative image features computed based on segmented tumors has begun to attract attention^29^. However, this was beyond the scope of our study. To maximally eliminate segmentation effects, we analyzed independent tumor measurements by three radiologists. Third, we only chose the most commonly used quantitative image features to study radiomic feature reproducibility. Despite these limitations, our findings appear to be robust and our study should increase awareness and understanding of the importance of proper imaging acquisition in the reproducible and reliable computation of quantitative image features in radiogenomics. That, in turn, should accelerate the identification of reliable and meaningful quantitative imaging biomarkers for tumors.

## Additional Information

**How to cite this article**: Zhao, B. *et al.* Reproducibility of radiomics for deciphering tumor phenotype with imaging. *Sci. Rep.* **6**, 23428; doi: 10.1038/srep23428 (2016).

## Supplementary Material

Supplementary Information

## Figures and Tables

**Figure 1:**
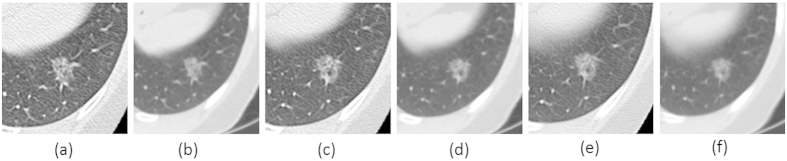
A lung tumor captured on one CT scan reconstructed at 6 different imaging settings: 1.25mm slice thickness with the lung reconstruction algorithm (sharp image) (1.25L) (**a**) and the standard reconstruction algorithm (smooth image) (1.25S) (**b**); 2.5mm slice thickness with lung reconstruction (2.5L) (**c**) and standard reconstruction (2.5S) (**d**); 5mm slice thickness with lung reconstruction (5L) (**e**) and standard reconstruction (5S) (**f**).

**Table 1:** Number (Percentage) of reproducible radiomic features at different CCC cut-offs.

	Repeat CT scan images reconstructed at								
	(a) same imaging setting						(b) different imaging settings		
CCC cut-off	1.25L1vs1.25L2	1.25S1vs1.25S2	2.5L1vs2.5L2	2.5S1vs2.5S2	5L1vs5L2	5S1vs5S2	1.25Lvs1.25S	2.5Lvs2.5S	5Lvs5S
0.95	35 (39)	46 (52)	34 (38)	44 (49)	23 (26)	34 (38)	3 (3)	3 (3)	6 (7)
0.90	67 (75)	73 (82)	57 (64)	68 (76)	49 (55)	56 (63)	8 (9)	10 (11)	11 (12)
0.85	83 (93)	78 (88)	68 (76)	79 (89)	66 (74)	72 (81)	17 (19)	17 (19)	16 (18)

The CCCs of the features were computed from repeat CT images reconstructed at (a) identical and (b) different imaging settings. There were 89 radiomic features in total.
