# Supplementary material for: Reproducibility of radiomics for deciphering tumor phenotype with imaging
Source: Sci Rep. 2016 Mar 24;6:23428. doi: 10.1038/srep23428 (PMC4806325; doi:10.1038/srep23428)
Supplement: Supplementary Information [file srep23428-s1.doc]

**Supplementary Information**

**Reproducibility of radiomics for deciphering tumor phenotype with imaging**

Binsheng Zhao^1,*^, Yongqiang Tan^1^, Wei-Yann Tsai^2^, Jing Qi^1^, Chuanmiao Xie^1^, Lin Lu^1^, Lawrence H. Schwartz^1^,

^1^Department of Radiology, Columbia University Medical Center, 630 West 168^th^ Street, New York, NY, 10032

^2^Department of Biostatistics, Columbia University Medical Center, 722 West 168^th^ Street, New York, NY, 10032

^*^Correspondence should be addressed to Binsheng Zhao (email: bz2166@cumc.columbia.edu).

**Supplementary Tables**

**Table S-1 CT imaging protocol**

| Scanning parameters | GE LightSpeed 16 (27/32) | GE VCT (4/32) |
| --- | --- | --- |
| Tube voltage | 120 kVp | 120 kVp |
| Tube current | 299-441 mA | 298-351 mA |
| Collimator configuration | 16 x 1.25 mm | 64 x 0.63 mm |
| Pitch | 1.375 | 0.984 |
| slice thickness | 1.25, 2.5 and 5mm | 1.25, 2.5 and 5mm |
| Reconstruction algorithm | Lung (L) and Standard (S) | Lung (L) and Standard (S) |

In this study, each patient underwent two unenhanced CT scans within 15 minutes. After the completion of the first scan, patients left the scanner table, walked around, and then laid back on the scanner table for a second scan. The scanners used were either a 16–detector row LightSpeed scanner, or a 64–detector row VCT scanner (GE Healthcare, Milwaukee, WI). During data collection, each of a patient’s two repeat scans was reconstructed into the 6 image series that were combinations of three slice thicknesses (1.25mm, 2.5mm and 5mm) and two reconstruction algorithms (lung (L) and standard (S)).

**Table S-2. Reproducibility / Agreement of radiomic features under multiple imaging settings**

|  | | | Repeat CT scan images reconstructed at | | | | | | | | |
| --- | --- | --- | --- | --- | --- | --- | --- | --- | --- | --- | --- |
|  |  |  | (**a**) same imaging setting | | | | | | (**b**) different imaging settings | | |
| No. | Feature | Dimension | 1.25L1vs1.25L2 | 1.25S1vs1.25S2 | 2.5L1vs2.5L2 | 2.5S1vs2.5S2 | 5L1vs5L2 | 5S1vs5S2 | 1.25Lvs1.25S | 2.5Lvs2.5S | 5Lvs5S |
| 1 | **Uni** | **1** | 0.99 | 0.99 | 0.98 | 0.99 | 0.98 | 0.99 | 0.99 | 0.99 | 0.98 |
| 2 | **Bi** | **2** | 0.99 | 0.99 | 0.99 | 0.99 | 0.99 | 0.99 | 0.99 | 0.99 | 0.99 |
| 3 | **Vol** | **3** | 1.00 | 1.00 | 1.00 | 1.00 | 1.00 | 1.00 | 1.00 | 1.00 | 1.00 |
| 4 | **Density_Mean** | **3** | 0.98 | 0.96 | 0.97 | 0.99 | 0.99 | 0.98 | 0.84 | 0.92 | 0.97 |
| 5 | **Density_SD** | **3** | 0.96 | 0.93 | 0.91 | 0.94 | 0.90 | 0.89 | 0.68 | 0.64 | 0.63 |
| 6 | **Density_Skewness** | **3** | 0.94 | 0.98 | 0.95 | 0.98 | 0.97 | 0.98 | 0.54 | 0.78 | 0.95 |
| 7 | **Density_Kurtorsis** | **3** | 0.94 | 0.98 | 0.81 | 0.98 | 0.93 | 0.97 | 0.71 | 0.80 | 0.92 |
| 8 | **Density_Mean_2D** | **2** | 0.94 | 0.95 | 0.84 | 0.94 | 0.96 | 0.88 | 0.81 | 0.86 | 0.92 |
| 9 | **Density_SD_2D** | **2** | 0.87 | 0.81 | 0.80 | 0.71 | 0.84 | 0.71 | 0.58 | 0.52 | 0.70 |
| 10 | **Density_Skewness_2D** | **2** | 0.87 | 0.93 | 0.79 | 0.95 | 0.92 | 0.90 | 0.33 | 0.60 | 0.84 |
| 11 | **Density_Kurtorsis_2D** | **2** | 0.89 | 0.85 | 0.81 | 0.95 | 0.86 | 0.92 | 0.45 | 0.74 | 0.87 |
| 12 | **Compact-Factor** | **3** | 0.86 | 0.92 | 0.94 | 0.95 | 0.91 | 0.91 | 0.88 | 0.93 | 0.84 |
| 13 | **Eccentricity_2D** | **2** | 0.84 | 0.87 | 0.89 | 0.86 | 0.84 | 0.75 | 0.88 | 0.84 | 0.75 |
| 14 | **Solidity_2D** | **2** | 0.59 | 0.82 | 0.70 | 0.74 | 0.72 | 0.60 | 0.80 | 0.69 | 0.69 |
| 15 | **Round-Factor_2D** | **2** | 0.53 | 0.68 | 0.78 | 0.63 | 0.82 | 0.85 | 0.64 | 0.59 | 0.78 |
| 16 | **Shape_Index2** | **3** | 0.89 | 0.90 | 0.89 | 0.74 | 0.84 | 0.84 | 0.77 | 0.79 | 0.66 |
| 17 | **Shape_Index3** | **3** | 0.94 | 0.94 | 0.95 | 0.88 | 0.89 | 0.92 | 0.78 | 0.80 | 0.76 |
| 18 | **Shape_Index4** | **3** | 0.94 | 0.97 | 0.93 | 0.94 | 0.92 | 0.91 | 0.81 | 0.81 | 0.81 |
| 19 | **Shape_Index5** | **3** | 0.95 | 0.96 | 0.95 | 0.98 | 0.90 | 0.94 | 0.79 | 0.84 | 0.77 |
| 20 | **Shape_Index6** | **3** | 0.91 | 0.94 | 0.88 | 0.92 | 0.91 | 0.89 | 0.87 | 0.82 | 0.76 |
| 21 | **Shape_Index7** | **3** | 0.96 | 0.96 | 0.95 | 0.98 | 0.91 | 0.91 | 0.93 | 0.90 | 0.82 |
| 22 | **Shape_Index8** | **3** | 0.95 | 0.89 | 0.90 | 0.91 | 0.87 | 0.85 | 0.71 | 0.69 | 0.62 |
| 23 | **Shape_Index9** | **3** | 0.99 | 0.97 | 0.96 | 0.98 | 0.96 | 0.95 | 0.91 | 0.89 | 0.85 |
| 24 | **Sigmoid-Amplitude** | **3** | 0.97 | 0.97 | 0.97 | 0.97 | 0.95 | 0.96 | 0.82 | 0.84 | 0.93 |
| 25 | **Sigmoid-Slope** | **3** | 0.91 | 0.91 | 0.95 | 0.95 | 0.91 | 0.93 | 0.15 | 0.21 | 0.29 |
| 26 | **Sigmoid-Offset** | **3** | 0.96 | 0.96 | 0.97 | 0.97 | 0.95 | 0.96 | 0.85 | 0.88 | 0.92 |
| 27 | **Wavelet_DWT-D** | **2.5** | 0.94 | 0.91 | 0.80 | 0.90 | 0.81 | 0.82 | 0.42 | 0.53 | 0.74 |
| 28 | **Wavelet_DWT-V** | **2.5** | 0.94 | 0.93 | 0.93 | 0.97 | 0.93 | 0.95 | 0.30 | 0.48 | 0.79 |
| 29 | **Wavelet_DWT-H** | **2.5** | 0.90 | 0.94 | 0.93 | 0.93 | 0.86 | 0.94 | 0.29 | 0.48 | 0.77 |
| 30 | **Wavelet_DWT-LD** | **2.5** | 0.88 | 0.83 | 0.77 | 0.77 | 0.60 | 0.76 | 0.13 | 0.30 | 0.36 |
| 31 | **Wavelet_DWT-LV** | **2.5** | 0.96 | 0.91 | 0.94 | 0.97 | 0.95 | 0.90 | 0.48 | 0.63 | 0.82 |
| 32 | **Wavelet_DWT-LH** | **2.5** | 0.92 | 0.96 | 0.93 | 0.91 | 0.89 | 0.84 | 0.59 | 0.75 | 0.78 |
| 33 | **EdgeFreq_Mean** | **2.5** | 0.93 | 0.97 | 0.93 | 0.96 | 0.95 | 0.98 | 0.15 | 0.19 | 0.25 |
| 34 | **EdgeFreq_Coarseness** | **2.5** | 0.99 | 0.96 | 0.99 | 0.99 | 0.81 | 0.95 | 0.93 | 0.90 | 0.76 |
| 35 | **EdgeFreq_Contrast** | **2.5** | 0.92 | 0.95 | 0.95 | 0.96 | 0.85 | 0.93 | 0.40 | 0.50 | 0.54 |
| 36 | **Fractal_Dimension-Mean** | **2.5** | 0.98 | 0.98 | 0.97 | 0.98 | 0.90 | 0.93 | 0.28 | 0.35 | 0.17 |
| 37 | **GTDM_Coarseness** | **2.5** | 0.98 | 0.95 | 0.98 | 0.98 | 0.93 | 0.97 | 0.79 | 0.78 | 0.70 |
| 38 | **GTDM_Contrast** | **2.5** | 0.96 | 0.90 | 0.94 | 0.93 | 0.88 | 0.96 | 0.67 | 0.66 | 0.61 |
| 39 | **GTDM_Busyness** | **2.5** | 0.99 | 0.91 | 0.99 | 0.95 | 0.98 | 0.97 | 0.85 | 0.80 | 0.74 |
| 40 | **GTDM_Complexity** | **2.5** | 0.89 | 0.85 | 0.84 | 0.77 | 0.88 | 0.83 | 0.04 | 0.05 | 0.07 |
| 41 | **GTDM_Strength** | **2.5** | 0.94 | 0.94 | 0.96 | 0.98 | 0.91 | 0.94 | 0.88 | 0.93 | 0.87 |
| 42 | **Gabor_Energy-sum** | **2.5** | 0.89 | 0.93 | 0.87 | 0.90 | 0.84 | 0.90 | 0.19 | 0.33 | 0.66 |
| 43 | **Gabor_Energy-dir0** | **2.5** | 0.81 | 0.95 | 0.87 | 0.83 | 0.88 | 0.87 | 0.42 | 0.61 | 0.82 |
| 44 | **Gabor_Energy-dir45** | **2.5** | 0.84 | 0.82 | 0.79 | 0.85 | 0.77 | 0.73 | 0.14 | 0.35 | 0.51 |
| 45 | **Gabor_Energy-dir90** | **2.5** | 0.91 | 0.93 | 0.87 | 0.88 | 0.84 | 0.82 | 0.18 | 0.30 | 0.56 |
| 46 | **Gabor_Energy-dir135** | **2.5** | 0.89 | 0.83 | 0.82 | 0.88 | 0.84 | 0.68 | 0.20 | 0.32 | 0.54 |
| 47 | **Laws_Energy-1** | **2.5** | 0.97 | 0.91 | 0.95 | 0.89 | 0.95 | 0.86 | 0.52 | 0.49 | 0.59 |
| 48 | **Laws_Energy-2** | **2.5** | 0.98 | 0.96 | 0.95 | 0.90 | 0.94 | 0.89 | 0.28 | 0.29 | 0.32 |
| 49 | **Laws_Energy-3** | **2.5** | 0.93 | 0.98 | 0.92 | 0.92 | 0.85 | 0.89 | 0.09 | 0.10 | 0.11 |
| 50 | **Laws_Energy-4** | **2.5** | 0.88 | 0.97 | 0.88 | 0.95 | 0.86 | 0.91 | 0.02 | 0.03 | 0.03 |
| 51 | **Laws_Energy-5** | **2.5** | 0.95 | 0.98 | 0.85 | 0.86 | 0.88 | 0.91 | 0.14 | 0.14 | 0.18 |
| 52 | **Laws_Energy-6** | **2.5** | 0.94 | 0.97 | 0.81 | 0.90 | 0.82 | 0.89 | 0.07 | 0.08 | 0.09 |
| 53 | **Laws_Energy-7** | **2.5** | 0.88 | 0.97 | 0.85 | 0.93 | 0.86 | 0.93 | 0.02 | 0.02 | 0.03 |
| 54 | **Laws_Energy-8** | **2.5** | 0.88 | 0.94 | 0.70 | 0.79 | 0.74 | 0.85 | 0.04 | 0.05 | 0.07 |
| 55 | **Laws_Energy-9** | **2.5** | 0.88 | 0.94 | 0.83 | 0.93 | 0.78 | 0.91 | 0.01 | 0.02 | 0.02 |
| 56 | **Laws_Energy-10** | **2.5** | 0.87 | 0.88 | 0.86 | 0.81 | 0.80 | 0.85 | 0.01 | 0.01 | 0.02 |
| 57 | **Laws_Energy-11** | **2.5** | 0.99 | 0.98 | 0.92 | 0.90 | 0.95 | 0.87 | 0.29 | 0.28 | 0.32 |
| 58 | **Laws_Energy-12** | **2.5** | 0.96 | 0.97 | 0.80 | 0.92 | 0.82 | 0.91 | 0.09 | 0.10 | 0.11 |
| 59 | **Laws_Energy-13** | **2.5** | 0.87 | 0.95 | 0.76 | 0.89 | 0.75 | 0.85 | 0.02 | 0.03 | 0.03 |
| 60 | **Laws_Energy-14** | **2.5** | 0.88 | 0.85 | 0.88 | 0.90 | 0.80 | 0.86 | 0.01 | 0.01 | 0.01 |
| 61 | **LoG_MGI-s1** | **2.5** | 0.98 | 0.91 | 0.94 | 0.97 | 0.95 | 0.95 | 0.86 | 0.85 | 0.83 |
| 62 | **LoG_Entropy-s1** | **2.5** | 0.94 | 0.97 | 0.92 | 0.98 | 0.93 | 0.97 | 0.11 | 0.16 | 0.21 |
| 63 | **LoG_Uniformity-s1** | **2.5** | 0.94 | 0.99 | 0.93 | 0.99 | 0.96 | 0.99 | 0.07 | 0.11 | 0.15 |
| 64 | **LoG_MGI-s4** | **2.5** | 0.99 | 0.94 | 0.96 | 0.98 | 0.97 | 0.97 | 0.88 | 0.88 | 0.85 |
| 65 | **LoG_Entropy-s4** | **2.5** | 0.99 | 0.97 | 0.99 | 0.99 | 0.97 | 0.98 | 0.90 | 0.90 | 0.88 |
| 66 | **LoG_Uniformity-s4** | **2.5** | 0.99 | 0.96 | 0.99 | 1.00 | 0.98 | 0.99 | 0.93 | 0.88 | 0.85 |
| 67 | **Run_SPE** | **3** | 0.94 | 0.99 | 0.97 | 0.99 | 0.98 | 0.99 | 0.12 | 0.18 | 0.28 |
| 68 | **Run_LPE** | **3** | 0.95 | 0.99 | 0.97 | 0.98 | 0.99 | 0.99 | 0.09 | 0.12 | 0.18 |
| 69 | **Run_GLU** | **3** | 0.99 | 0.80 | 0.99 | 1.00 | 0.94 | 1.00 | 0.86 | 0.93 | 0.77 |
| 70 | **Run_PLU** | **3** | 0.98 | 0.64 | 0.99 | 0.99 | 0.95 | 0.99 | 0.51 | 0.30 | 0.29 |
| 71 | **Run_PP** | **3** | 0.94 | 0.99 | 0.97 | 0.99 | 0.98 | 0.99 | 0.12 | 0.17 | 0.26 |
| 72 | **Spatial_Corr** | **2.5** | 0.95 | 0.94 | 0.86 | 0.93 | 0.89 | 0.96 | 0.12 | 0.20 | 0.46 |
| 73 | **GLCM_ASM** | **3** | 0.93 | 0.98 | 0.96 | 0.98 | 0.95 | 0.98 | 0.07 | 0.11 | 0.16 |
| 74 | **GLCM_Contrast** | **3** | 0.93 | 0.93 | 0.92 | 0.92 | 0.91 | 0.94 | 0.23 | 0.23 | 0.24 |
| 75 | **GLCM_Corr** | **3** | 0.93 | 0.84 | 0.78 | 0.90 | 0.83 | 0.87 | 0.10 | 0.10 | 0.26 |
| 76 | **GLCM_Sum-Squares** | **3** | 0.97 | 0.91 | 0.90 | 0.93 | 0.93 | 0.87 | 0.63 | 0.57 | 0.59 |
| 77 | **GLCM_Homogeneity** | **3** | 0.94 | 0.99 | 0.96 | 0.99 | 0.97 | 0.99 | 0.17 | 0.23 | 0.32 |
| 78 | **GLCM_IDM** | **3** | 0.93 | 0.99 | 0.96 | 0.99 | 0.97 | 0.99 | 0.13 | 0.20 | 0.30 |
| 79 | **GLCM_Sum-Average** | **3** | 0.98 | 0.96 | 0.97 | 0.99 | 0.99 | 0.99 | 0.86 | 0.94 | 0.98 |
| 80 | **GLCM_Sum-Variance** | **3** | 0.98 | 0.97 | 0.97 | 0.99 | 0.99 | 0.99 | 0.83 | 0.93 | 0.97 |
| 81 | **GLCM_Sum-Entropy** | **3** | 0.97 | 0.98 | 0.96 | 0.98 | 0.96 | 0.98 | 0.62 | 0.66 | 0.72 |
| 82 | **GLCM_Entropy** | **3** | 0.95 | 0.98 | 0.96 | 0.98 | 0.95 | 0.98 | 0.33 | 0.39 | 0.48 |
| 83 | **GLCM_Diff-Variance** | **3** | 0.95 | 0.99 | 0.96 | 0.99 | 0.97 | 0.99 | 0.21 | 0.29 | 0.38 |
| 84 | **GLCM_Diff-Entropy** | **3** | 0.95 | 0.98 | 0.96 | 0.98 | 0.96 | 0.98 | 0.27 | 0.33 | 0.38 |
| 85 | **GLCM_IMC1** | **3** | 0.98 | 0.91 | 0.94 | 0.93 | 0.64 | 0.80 | 0.23 | 0.10 | 0.00 |
| 86 | **GLCM_IMC2** | **3** | 0.99 | 0.96 | 0.96 | 0.96 | 0.87 | 0.77 | 0.37 | 0.30 | 0.15 |
| 87 | **GLCM_MCC** | **3** | 0.85 | 0.80 | 0.60 | 0.70 | 0.65 | 0.83 | 0.05 | 0.05 | 0.21 |
| 88 | **GLCM_Max-Prob** | **3** | 0.90 | 0.96 | 0.93 | 0.96 | 0.89 | 0.97 | 0.05 | 0.08 | 0.12 |
| 89 | **GLCM_Cluster-Tendency** | **3** | 0.97 | 0.91 | 0.88 | 0.93 | 0.92 | 0.86 | 0.71 | 0.64 | 0.64 |

**Table S-2.** Agreement between radiomic features generated under multiple imaging settings.

The CCCs of the radiomic features computed for repeat CT scans (first vs second) reconstructed at (a) identical and (b) different imaging settings. Pink-highlighted cells indicate features for which CCC ≥ 0.85 at a given imaging setting of the repeat CT scan images. The dimension column shows how the features were computed, in 2D, 2.5D or 3D.

**Supplementary Methods**

**Definitions of Radiomic Features**

In this work, we studied a well-defined, comprehensive set of 89 radiomic features. Some features were computed in 3D, some were in 2D, and some were in both. Several feature classes were defined in 2.5D, i.e., features were first computed on each 2D image and then combined into 3D features by averaging of 2D features. The 2D features were calculated on the automatically determined axial image where the lesion had the maximal diameter. Hereafter this image will be called the 2D image. Eight connected pixels were considered as neighboring pixels for 2D analysis, whereas 26 connected voxels were considered as the neighboring voxels for 3D analysis. Eight directions were used for 2D analysis and 13 directions were used for 3D analysis. Unless specified, the distance *d* between two neighboring pixels was one (1) when computing texture features.

The 89 radiomic features were grouped into 15 feature classes. A brief illustration of each feature is given below. In the following text, we use tumor and object interchangeably, and density, gray-tone and gray-level interchangeably. The location of a pixel (2D) or a voxel (3D) in an image array is represented as (x, y) or (x, y, z).  For simplicity, *i* is sometimes used to represent an image element. The image gray-level is correspondingly denoted as f(x,y), f(x,y,z) or f(i). Those interested in a more detailed explanation of any particular feature or feature class are referred to relevant publications.

**Feature class #1.** Size-related (feature numbers: 1-3)

An object's size can be quantified by its diameter, area and volume. In this work, uni-dimensional (Uni), bi-dimensional (Bi) and volumetric measurements were calculated for each tumor. Uni (maximal diameter) was measured by counting the number of pixels in the longest line across the tumor on the 2D image multiplied by the image spatial resolution in the x- (or y-) direction. Bi was defined as the product of the maximal diameter and its maximal perpendicular diameter, measured by counting the number of pixels in the longest line perpendicular to the maximal diameter on the 2D image multiplied by the image spatial resolution in the x- (or y-) direction. Volume (Vol) was calculated by multiplying the number of tumor voxels by the image spatial resolutions in the x-, y- and z-directions, respectively.

**Feature class #2.** First Order Statistics (feature numbers: 4 - 11)

First-order statistics features are histogram-related. We chose 4 commonly used first-order statistics features to describe tumor density distribution without any information about their spatial arrangements. Since these features were calculated in both 2D and 3D, there were a total of 8 features in this class.

Let
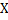
(i) denote the density of the i_th_ element in an image matrix that has
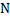
 voxels (3D) / pixels (2D), the 4 computed first-order statistics features are:

***Density_Mean:***


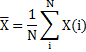
 (1)

***Density_SD:***


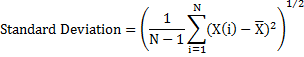
(2)

***Density_Skewness:***


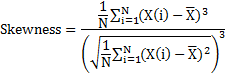
(3)

***Density_Kurtorsis:***


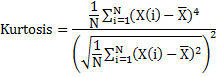
(4)

Skewness is a measure of the degree of distribution symmetry. A value of zero (0) indicates that the distribution has a normal shape; a positive value indicates a shift of the distribution peak from the center to its left, whereas a negative value implies a shift of the distribution peak from the center to its right.

Kurtosis is a measure of whether a distribution is peaked or flat related to a normal shape. A value of 3 means the distribution has a normal shape; a larger value indicates a peaked distribution corresponding to a more homogenous data set, whereas a smaller value implies a flat distribution corresponding to a more heterogeneous dataset.

**Feature Class #3.** Shape (feature numbers: 12 - 15)

Shape-related features describe the shape properties of an object.

Compact-Factor (CF) quantifies the compactness of a tumor in 3D. Let *V* denote tumor volume and *S* denote tumor surface. In this work, CF was defined as


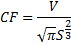
 (5)

The closer the value of CF to 1, the more compact the tumor.

Eccentricity is a measure specifying how close an ellipse is to a circle. We used the following definition for eccentricity:

*Eccentricity = c / a* (6)

where, *c* is the distance from the center to a focus and *a* is the distance from that focus to a vertex. It's a 2D feature. A line shape has a value of one (1) and a circle has a value of zero (0).

Round-Factor (RF) is a measure of the roundness of a 2D object. Let *Area* denote the area of the tumor and *Perimeter* the length of the tumor contour on the reference image.


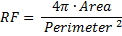
 (7)

The closer the value of RF to one (1) is, the rounder the object will be.

Solidity is the ratio of the object area over the area of the convex hull bounding the object. Let *Area* denote the object’s area and *ConvexArea* the area of the convex hull bounding the object on the reference image. Solidity is defined as:


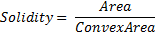
 (8)

Solidity has a maximal value of one (1) when the object shows a convex shape.

**Feature Class #4.** Surface Shape (feature numbers: 16 - 23)

The Shape Index features capture the intuitive notion of 'local surface shape' of a 3D object . Let
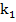
 and
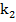
 denote the two principal curvatures of a point on the surface. Shape index is then defined as


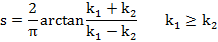
 (9)

The shape index is a numerical value within the range of [-1,1] and can be divided into 9 categories, each representing one of the following 9 shapes, spherical cup, trough, rut, saddle rut, saddle, saddle ridge, ridge, dome and spherical cap (Fig. S-1).

Shape_SI1: spherical cup, value of
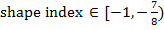
 .

Shape_SI2: trough, value of
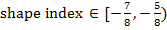
 .

Shape_SI3: rut, value of
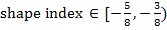
 .

Shape_SI4: saddle rut, value of
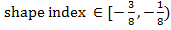
 .

Shape_SI5: saddle, value of
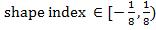
 .

Shape_SI6: saddle ridge, value of
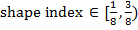
 .

Shape_SI7: ridge, value of
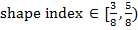


Shape_SI8: dome, value of
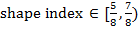


Shape_SI9: spherical cap, value of
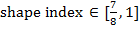
.

We adopted the algorithm proposed by Thiron to compute the two principal curvatures
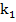
 and
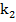
. In Thiron’s algorithm,
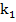
 and
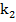
 are the solutions of an equation of order two,


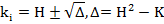
 (10)

where
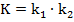
 is the Gaussian curvature and
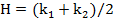
 is the mean curvature.

Based on the implicit function theorem, Thiron proposed two formulations for the computation of
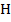
 and
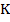
, which only make use of differentials of the 3D surface.


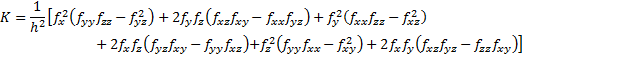
(11)


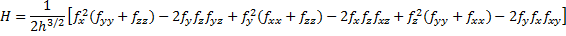
 (12)

where
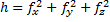
;
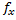
,
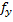
 and
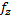
 are the 3 first derivatives;
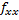
,
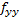
,
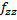
,
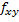
,
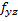
and
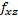
 are the 6 second derivatives.


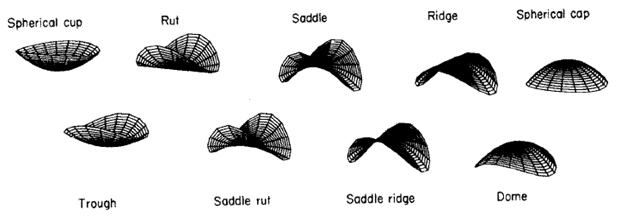


(SI 1)

(SI 3)

(SI 7)

(SI 2)

(SI 4)

(SI 6)

(SI 8)

(SI 9)

(a) Nine surface shapes

(b) Shape Index scales [-1, 1]

Figure S-1 Surface shapes and Shape Index features (cited from literature ).

The sum of the 9 scaled shape indexes equals 1, i.e.,
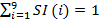
. To reduce the redundancy, we excluded the first Shape Index from the analysis.

**Feature Class #5.** Sigmoid Functions (feature numbers: 24-26)

To quantify the density relationship between a tumor and its surrounding background of lung parenchyma, e.g., sharpness of the tumor margin, we applied sigmoid curves to fit density changes along sampling lines drawn orthogonal to the tumor surface. Each sampling line went through one voxel on the tumor surface with 5mm inside and 5 mm outside the tumor.

The sigmoid curve function is defined as,


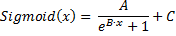
 (13)

where the fitting parameter *A* specifies the amplitude of the curve, *B* specifies the slope of the curve (from the tumor to its background) and *C* is the offset of the curve. The three Sigmoid Function features we studied were the average values of the three fitting parameters of *A*, *B* and *C* over all surface voxels.

Sigmoid-Amplitude: average of the amplitude values (A) of all sampling lines.

Sigmoid-Slope: average of the slope values (B) of all sampling lines.

Sigmoid-Offset: average of the offset values (C) of all sampling lines.

**Feature Class #6.** Wavelets features (feature numbers: 27-32)

The discrete wavelet transform (DWT) was chosen as one means to analyze tumor coarse and fine structures. Taking a *P*=*M* x *N* image *I*(*m, n*) as an example, the first level DWT decomposition can be briefly described as the following. First, a low-pass and a high-pass filter (a ‘Coiflets1’ wavelet filter was used in this study) are applied to the original image vertically, followed by a vertical down-sampling by a factor of 2. Then, the two filters are applied to the processed image horizontally followed by a horizontal down-sampling by a factor of 2. This results in 4 sub-images that are known as the low-pass approximation *L*(*m, n*) (also called average image), vertical detail *V(m,n)*, horizontal detail *H*(*m, n*) and diagonal detail *D*(*m, n*). The second level DWT decomposition repeats the above procedure but with the average image generated at the first level decomposition. Figure 2 shows an example of a two level DWT decomposed lung image.


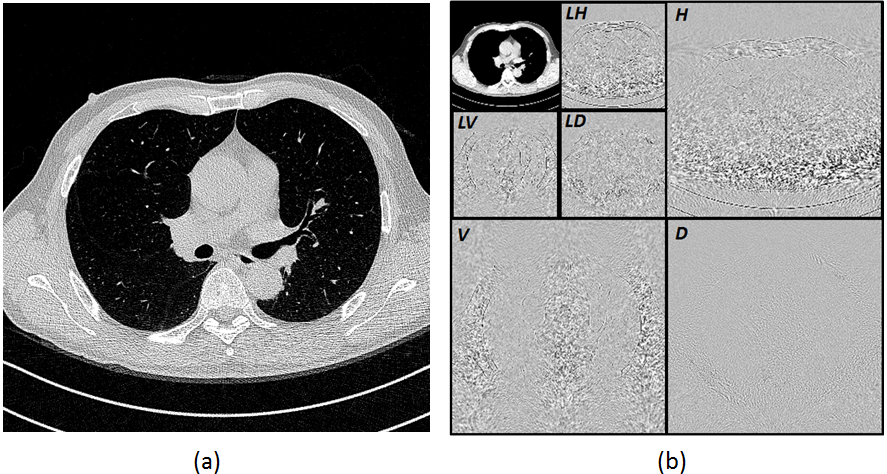


Figure S-2. (a) Original 2D image of a lung. (b) The DWT decomposed sub-images at two levels / scales.

In this study, the 7 wavelet features were defined as the Energy of each detailed sub-image.

At the first DWT decomposition level,

***DWT-H:***

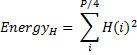
 (14)

***DWT-V:***


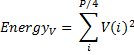
 (15)

***DWT-D:***


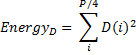
 (16)

At the second DWT decomposition level,

***DWT-LH:***


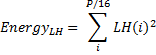
 (17)

***DWT-LV:***


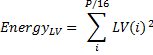
 (18)

***DWT-LD:***

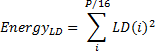
 (19)

**Feature Class #7.** Edge Frequency features (feature numbers: 33 - 35)

Edge Frequency features characterize variation of the density gradients of a tumor . We chose Robert's edge operator to transform the original image into a gradient image, and extracted the mean, coarseness and contrast features based on the gradient image.

For a 2D image, the Robert's edge operator is defined as follows:


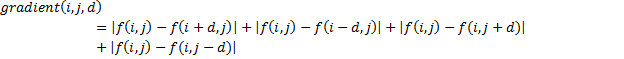
(17)

where
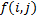
 denotes an object pixel density at the location (*i, j*), and
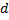
 is the distance between the pixel
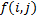
 and its neighboring pixel. We extracted the following three features in this class.

***EdgeFreq_Mean*:** average of the gradients over the tumor.

***EdgeFreq_Coarseness*:** coarseness of the gradient image.

***EdgeFreq_Contrast:*** contrast of the gradient image.

The equations to compute Coarseness and Contrast are the same as the ones defined in Feature Class #8 GTDM. We replace *S*(*i*) with gradient(*d*).

**Feature Class #8.** Fractal Dimension (Feature number: 36)

Fractal dimension provides a statistical index for quantifying the complexity of an image . Basically, the fractal dimension describes the relationship between the changes in a measuring scale and the resultant measurement value at the scale. The rougher the texture, the larger the fractal dimension.

In this study, a 3D box-counting algorithm was adopted to calculate the fractal dimension of a tumor . Supposing the X-Y side of the box corresponds to a 2D image, and its orthogonal direction of Z represents image density, the calculation of the fractal dimension to quantify the density distribution of an image at a certain scale
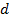
 can be illustrated by the following steps.

*Step1:* choose the range of the measuring scales
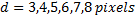
.

*Step2:* at each scale
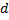
, apply a sliding window of
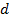

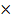

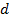
 pixels over the tumor. In each window, compute as follows:


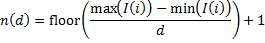
 (18)

Where
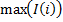
 and
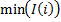
 are the maximum and minimum image densities inside the window. The floor function is the greatest integer function.

The total number of boxes to cover the tumor density is defined as,


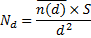
 (19)

where
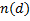
 is the mean value of all
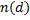
 and *S* is the tumor area

*Step3:* repeat step 2 for each
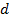
.

*Step4:* calculate the fractal dimension (
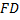
) as the slope of the regression line of the following equation by the least-squares methods.


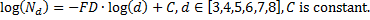
 (20)

**Feature Class #9.** Gray-Tone Difference Matrix (GTDM) (feature numbers: 37 - 41)

Neighborhood GDTM features describe visual properties of texture based on gray-tone difference between a pixel and its neighborhood . The computation of GDTM features is illustrated as follows. Let
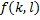
 be an image pixel that has the gray-tone of
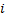
 and is located at
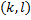
. The average gray-tone over a neighborhood centered at, but excluding
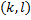
, is


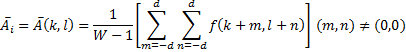
 (21)

where (2*d* + 1) is the neighborhood size and
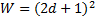
.

The
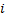
th entry in the GDTM is


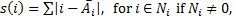


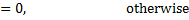
 (22)

where
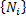
 is the set of all pixels having the gray tone of
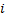
.

Thus, for an
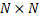
 image, let
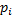
 denote the probability of occurrence of gray-tone value *i*,
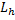
 denote the highest gray-tone value present in the image and
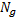
 denote the total number of different gray-tone values present in the image. The GDTM features are defined as,

***Coarseness:***


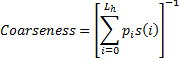
 (23)

***Contrast:***


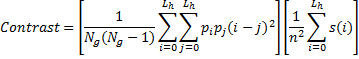
 (24)

***Busyness:***


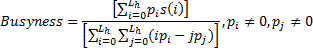
 (25)

***Complexity:***


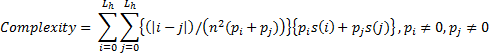
 (26)

***Strength:***


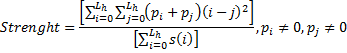
 (27)

In this study, we normalized an image into 256 density bins so that
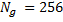
. The distance between neighboring pixels was set to 1.

**Feature Class #10.** Gabor Energy (feature numbers: 42 - 46)

Gabor filters are linear filters designed for edge detection, which are used in image processing for feature extraction and texture analysis . For a 2D image, the Gabor filter is defined as


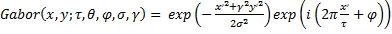
 (28)

where
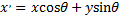
 and
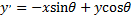
.
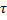
 is the wavelength of the sinusoidal factor,
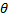
 is the orientation of the normal to the parallel lines of a Gabor function,
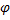
 is the phase offset,
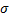
 is the sigma of the Gaussian function and
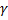
 is the spatial aspect ratio.

In this study, wavelength was set to pixels. The other parameters were: , and =1.0. Gabor features were computed from Gabor Energy that is defined as

(29)

where *Gabor*(*i*) was a Gabor filter processed (original) image and *N* was the number of tumor pixels. The Gabor feature class included 5 features, i.e., the Energy calculated in 4 directions plus an average of all directions.

**Feature Class #11.** Laws' Energy (feature numbers: 47 - 60)

Laws' Energy features emphasize image textures of edge, spot, ripple and wave through the Laws filters built by the following 5 basic vectors .

Average: (30)

Edge: (31)

Spot: (32)

Ripple: (33)

Wave: (34)

By multiplying the transpose of the vector and/or the vector itself (e.g., ), 14 standard Laws filters can be created as listed below:

Laws filter #1: + ;

Laws filter #2: + ;

Laws filter #3: + ;

Laws filter #4: + ;

Laws filter #5: + ;

Laws filter #6: + ;

Laws filter #7: + ;

Laws filter #8: + ;

Laws filter #9: + ;

Laws filter #10: + ;

Laws filter #11: ;

Laws filter #12: ;

Laws filter #13: ;

Laws filter #14: .

Each Laws filter enhances one of the above-mentioned patterns along x and y directions. Laws filter #1 enhances edges in both directions. The Laws' Energy features #1 - #14 are the energies computed from the pre-processed original images by Laws' filters #1 - #14, respectively,

(35)

where is the number of object pixels.

**Feature Class #12.** Laplacian of Gaussian (LoG) (feature numbers: 61 - 66)

The Laplacian is a differential operator that can be used to highlight regions of rapid gray-level change in images. Because of its sensitivity to image noise, a Gaussian smoothing filter is applied beforehand to reduce noise. The combined filter is called Laplacian of Gaussian (LoG) .

The definition of a 2D LoG is:

, (36)

The texture at different scales (fine to coarse) is highlighted by varying Gaussian kernels (. The smaller the the finer the texture that can be described.

In this study, the following three LoG features of mean, uniformity and entropy were calculated from the LoG filtered (original) image, , at (s1; no smoothing) and (s4).

***LoG Mean Gray Intensity (MGI):***

(37)

***LoG Uniformity:***

(38)

***LoG entropy:***

(39)

where is the number of object pixels, is the probability of pixels with a gray-level of in the LoG pre-processed image, and is the maximal value of the pre-processed image.

**Feature Class #13.** Run-Length features (feature numbers: 67 - 71)

The run-length features are used to characterize image coarseness by counting the number of maximum contiguous pixels / voxels having a constant gray-level along a line . A larger number of neighboring pixels of the same gray-level represents a coarser texture, whereas a smaller number of neighboring pixels indicates a finer texture.

Let be the number of primitives of all directions having length and gray-level , the tumor volume, the maximum run-length, and the number of image gray-levels. Thus, the total number of run-lengths is

(40)

Then, the five run-length features we used are

***Run_SPE****:* Short primitives emphasis

(41)

***Run_LPE***: Long primitives emphasis

(42)

***Run_GLU***: Gray-level uniformity

(43)

***Run_PLU*:** Primitive length uniformity

(44)

***Run_PP***: Primitive percentage

(45)

**Feature Class #14.** Spatial Correlation (feature number: 72)

Spatial correlation features assess linear spatial relationships between texture primitives (a single pixel / voxel here) . The value of spatial correlation feature decreases slowly with increasing distance for a coarse texture, whereas it decreases rapidly for a fine texture.

Let be an image pixel's gray-level at the location (x, y) in a tumor, the distance between two pixels, the area of the tumor, the area of the tumor after shrinking with a distance of pixels. Then,

(46)

In this study, the spatial correlation was calculated at =1 pixel.

**Feature Class #15.** Gray-Level Co-occurrence Matrix (GLCM) (feature numbers: 73 - 89)

This class of features characterizes the textures of an image / object by creating a new matrix GLCM based on counting how often pairs of pixels with specific gray-level values and in a specified spatial relationship (distance and direction) occur in the image / object, and then computing statistics from the GLCM .

A GLCM is defined as , a matrix with a size of describing how often a pixel with gray value occurs adjacent to a pixel with the value . The two pixels are separated by a distance of pixels in the direction of . is the number of gray-level bins. Figure S-3 shows the procedure of constructing GLCM and computing its Homogeneity and Contrast features on a 2D example image.

Figure S-3. Construction of a GLCM and extraction of Homogeneity and Contrast features on a 2D example image

(a) An example of a gray-level image. (b) Pixel gray values of the image, ranging from 1 to 4. (c) A GLCM derived from the original image at and . (d) Extraction of Homogeneity and Contrast features based on a GLCM.

In this study, the number of gray value bins () was set to 256, distance was 1 and each feature was calculated for 13 directions in 3D. The average of the 13 measures of each feature was used as the final measure for that feature. We extracted 17 standard GLCM features as defined below.

***Angular Second Moment (ASM):***

(47)

***Contrast:***

(48)

***Correlation (Corr):***

(49)

***Sum of squares:***

(50)

***Homogeneity:***

(51)

***Inverse Difference Moment (IDM):***

(52)

***Sum average (SA):***

(53)

***Sum entropy (SE):***

(54)

***Sum variance (SV):***

(55)

***Entropy:***

(56)

***Different Variance (DV):***

(57)

***Different Entropy (DE):***

(58)

***Informational measure of correlation 1 (IMC1):***

(59)

***Informational measure of correlation 2 (IMC2):***

(60)

***Maximum Correlation Coefficient (MCC):***

(61)

(62)

***Maximal Probability (MP):***

(63)

***Cluster Tendency (CT):***

(64)

where:

: the probability distribution matrix of co-occurrence matrix ,

: the number of discrete intensity levels in the image,

: the mean of ,

is the marginal row probabilities,

is the marginal column probabilities,

: the mean of ,

: the mean of ,

: the standard deviation of ,

: the standard deviation of ,

, , ,

, , ,

is the entropy of ,

is the entropy of ,

is the entropy of ,

,

.

**References**

1. Jan, J.K. and J.D. Andrea, *Surface shape and curvature scales.* Image and Vision Computing, 1992. **10**(8): p. 557-565.

2. Thirion, J.P. and A. Gourdon, *Computing the differential characteristics of isointensity surfaces.* Computer vision and image understanding, 1995. **61**(2): p. 190-202.

3. Napel, S.A., et al., *Automated retrieval of CT images of liver lesions on the basis of image similarity: method and preliminary results.* Radiology, 2010. **256**(1): p. 243-52.

4. Davis, L.S. and A. Mitiche, *Edge detection in textures.* Computer Graphics and Image Processing, 1980. **12**(1): p. 25-39.

5. Mandelbrot, B., *How long is the coast of Britain? Statistical self-similarity and fractional dimension.* Science, 1967. **156**(3775): p. 636-638.

6. Kido, S., et al., *Fractal analysis of internal and peripheral textures of small peripheral bronchogenic carcinomas in thin-section computed tomography: comparison of bronchioloalveolar cell carcinomas with nonbronchioloalveolar cell carcinomas.* J Comput Assist Tomogr, 2003. **27**(1): p. 56-61.

7. Amadasun, M., *Texural Features Corresponding to Texural Properties.* IEEE Transactions on Systems, Man and Cybernetics, 1989. **19**(5): p. 1264-1274.

8. Fogel, I. and D. Sagi, *Gabor filters as texture discriminator.* Biological Cybernetics, 1989. **61**(2): p. 103-113.

9. Laws, K.I. *Rapid Texture Identification*. in *Image Processing for Missile Guidance*. 1980. San Diego: SPIE.

10. Ganeshan, B., et al., *Tumour heterogeneity in oesophageal cancer assessed by CT texture analysis: preliminary evidence of an association with tumour metabolism, stage, and survival.* Clin Radiol, 2012. **67**(2): p. 157-64.

11. Galloway, M.M., *Texture analysis using gray level run lengths.* Computer Graphics and Image Processing, 1975. **4**(2): p. 172-179.

12. Castleman, K.R., *Digital Image Prncessing*, ed. E. Cliffs. 1996, NJ: Prentice-Hall.

13. Haralick, R.M., K. Shanmugam, and I. Dinstein, *Textural Features for Image Classification.* IEEE Transactions on Systems, Man and Cybernetics, 1973. **3**(6): p. 610-621.
